# Sleep and mood in central serous chorioretinopathy

**DOI:** 10.1038/s41433-025-03688-3

**Published:** 2025-02-26

**Authors:** Yunfei Yang, Victoria S. Foster, Sophie Marlowe, Sarah R. Stevenson, Iona Alexander, SOMNUS Study Group, Susan Downes, Rukhsana Safa, Katharina Wulff, Iona Alexander, Sophie Marlowe, Colm Andrews, Caroline Justice, Alexina Fantato, Russell Foster, Hiten Sheth, Judith Abrams, Katarina Manso, Rupert Bourne, Paula Turnbull, Anthony Moore, Phil Hykin, Emily Summers, Muhammed Tahir, Sue Nuth, Emma Craig, Amanda Churchill, Eleanor Hiscott, Graeme Black, James Kirwan, Mini David, Marcela Votruba, Geeta Menon, Ganga Pathinayake, Nora Mistersky, Russell G. Foster, Susan M. Downes

**Affiliations:** 1 Department of Ophthalmology, Lister Hospital, East and North Hertfordshire NHS Foundation Trust, Coreys Mill Ln, Stevenage, SG1 4AB England; 2 Oxford Eye Hospital, West Wing, Oxford University Hospitals Foundation Trust, Headington, Oxford, OX3 9DU England; 3 St George’s, University of London, Medical School, Cranmer Terrace, London, SW17 0RE UK; 4 Great Ormond Street Hospital, Great Ormond Street, London, WC1N 3JH UK; 5 Ophthalmology Department of the Royal Gwent Hospital, Cardiff Road, Newport, NP20 2UB UK; 6 Sir Jules Thorn Sleep and Circadian Neuroscience Institute, Nuffield Department of Clinical Neurosciences, Dorothy Crowfoot Hodgkin Building, University of Oxford, South Parks Road, Oxford, OX1 3QU England; 7 Nuffield Laboratory of Ophthalmology, Nuffield Department of Clinical Neurosciences, Dorothy Crowfoot Hodgkin Building, University of Oxford, South Parks Road, Oxford, OX1 3QU England; 8 Oxford University Hospitals NHS Trust and Nuffield Laboratory of Ophthalmology, University of Oxford, Oxford, England; 9 Buckinghamshire Healthcare NHS Trust, Buckinghamshire, UK; 10 Hinchinbrook Hospital, Huntingdon, UK; 11 Moorfields Eye Hospital, London, UK; 12 Royal Berkshire NHS Foundation Trust, Reading, UK; 13 Bristol Eye Hospital, Bristol, UK; 14 Central Manchester University Hospitals NHS Foundation Trust, Manchester, UK; 15 Portsmouth Hospitals NHS Trust, Cosham, UK; 16 University Hospital Wales, Cardiff, UK; 17 Frimley Health NHS Foundation Trust, Camberley, UK

**Keywords:** Retinal diseases, Neuroscience

## Abstract

**Purpose:**

To investigate the impact of central serous chorioretinopathy on sleep and mood in patients with acute and chronic central serous chorioretinopathy.

**Methods:**

This cross-sectional study compared sleep and mood differences between central serous chorioretinopathy and control patients recruited from Ophthalmology clinics at the John Radcliffe Hospital, Oxford between 2012 and 2020. Data including visual acuity, type of central serous chorioretinopathy (acute or chronic; aCSC/cCSC), sex, and chronotype were obtained. Sleep quality was measured using the Pittsburgh Sleep Quality Index (PSQI); the Hospital Anxiety and Depression Scale (HADS) was used to evaluate anxiety (HADS-A) and depression (HADS-D).

**Results:**

A total of 247 age matched controls and 109 patients with central serous chorioretinopathy participated. There were no significant differences in PSQI or HADs (*P* > 0.05) between the two groups. Females exhibited significantly higher PSQI scores than males both for control and central serous chorioretinopathy groups (*P* < 0.05). Within the central serous chorioretinopathy group, 88 (81%) had chronic central serous chorioretinopathy and 21 (19%) had acute central serous chorioretinopathy, and an increase in daytime dysfunction was seen in the acute phenotype compared to chronic (*P* = 0.018).

**Conclusion:**

In our study, no significant differences in sleep quality or mood scores were identified in central serous chorioretinopathy patients, when compared to controls. Worsened sleep for females was present when compared to males, both in central serous chorioretinopathy and control groups. Within central serous chorioretinopathy groups, worsened daytime function was observed in acute versus chronic – a larger study would help distinguish the effect of chronicity on sleep.

## Introduction

Patients with visual impairment or visual loss are reported to have an increased incidence of sleep disorders [1, 2]. This has been reported in anophthalmic patients (those without eyes either congenital anophthalmos or loss of an eye due to enucleation as a consequence of trauma, or disease). It has also been reported in individuals with visual impairment due to different ophthalmic conditions [3–8]. Damage to retinal tissue has the potential to cause downstream effects on cell populations within the retina, other than the photoreceptors associated with vision, namely the photosensitive retinal ganglion cells (pRGCs) [9–12]. The pRGC population assists in synchronisation of circadian rhythms, such as the sleep-wake cycle, through their perception of the dawn/dusk cycle [13]. In the absence of light input to the suprachiasmatic nuclei (SCN), circadian rhythms become uncoupled from time cues (zeitgebers) in relation to the environment – the strongest of which is light. However, behaviours such as irregular exposure to external light (and in turn, a lack of opportunity to experience zeitgebers in the external environment) may also have an impact on sleep disruption.

Indeed, removal of cataracts has been shown to improve sleep quality, potentially due to improved access of light to the pRGCs [14–16]. Sleep and circadian rhythm disruption (SCRD) can have a detrimental impact on health and well-being. SCRD has been described in multiple ocular conditions, in particular in bilateral eye loss either congenital or acquired [7, 8, 17], but also including age related macular degeneration (AMD) [18], glaucoma [19] and inherited retinal degeneration (IRD) [20].

In this study, we investigate whether central serous chorioretinopathy (CSC) is associated with SCRD. CSC has an incidence of 9.9 cases per 100,000 men and 1.7 per 100,000 women [21–23] and is characterised by serous fluid collecting in the subretinal space. Symptoms such as blurring, distortion, fluctuating vision, image metamorphopsia, or scotomata in chronic cases become apparent if the disease involves the macula [24]. In some cases of CSC, retinal damage can occur and may lead to visual impairment. If this retinal damage also involves pRGCs and their function is affected, this might also contribute to SCRD.

CSC is generally classified into two groups: acute (aCSC) and chronic (cCSC). aCSC is usually self-limiting, with a sudden onset and short duration (averaging 2–4 months), and tends to present in younger age groups with a male preponderance [22, 25]. It can resolve fully but can recur [22, 25]. Up to 90% of aCSC cases resolve without intervention [26]. By contrast, cCSC is seen in older age groups, and is associated with progressive disease and visual loss [24, 27]. Bilateral findings of diffuse, multifocal leakage on retinal angiography with atrophy are seen in longstanding cases. Complications in cCSC such as persistent serous retinal detachment, cystoid macular degeneration, retinal pigment epithelium (RPE) decompensation or choroidal neovascularisation can lead to deterioration in central vision [28, 29]. Currently management of this condition is either conservative including assessment and addressing of potential risk factors (highlighted in more detail below) such as steroid use, hypertension, and stress or interventional using laser such as photodynamic therapy [30]. Focal thermocoagulation laser may be used if there is a discrete focal leak away from the fovea. In some cases where the CSC is complicated by choroidal neovascularisation, intravitreal anti-vascular endothelial growth factor agents have been used [31]. Eplenerone, a mineralocorticoid receptor antagonist was shown to be ineffective in the VICI trial [32], and micropulse laser therapy was shown to be inferior to photodynamic therapy in the PLACE trial [29, 30, 33–35].

Several potential risk factors for developing CSC have been described and include the following: genetic predisposition [31] medication (including antibiotics, psycho-pharmacologics) [36] and corticosteroid exposure [37–39], Type A personality [40–42], shift work [43], increased sympathetic activity [44], psychological stress with poor stress coping strategies [36, 41], blood pressure elevation [45], smoking [46] and reflux with *Helicobacter pylori* [47] have all been described. In addition, a strong association between post-traumatic stress disorder (PTSD) and development of CSC has been seen in veterans [48]; which may be due in part to malfunction of the stress axis as CSC is also linked to impaired autonomic regulation [49, 50].

As reported in association with other ocular conditions [51], CSC has been associated with obstructive sleep apnoea (OSA), a known sleep disruptive pathology [52–56]. One prior research article reported worsened sleep in CSC patients even whilst excluding individuals with OSA [57]. This suggests another underlying driver of poor sleep, however, this study did not control for shift work, chronotype, or subcategorise between aCSC and cCSC participants.

The impact of CSC on sleep and mood has only been investigated in one previous study [57], and never before in a UK population. In our questionnaire-based study we explored the effect of CSC on sleep quality and mood, controlling confounders where possible. In addition, we analysed whether acute and chronic disease may have a different impact on an individual’s sleep and mood. Finally, we also studied daytime sleepiness, chronotype and diurnal preference as well as the effects of an individual’s sex.

## Methods

### Demographics

A total of 109 participants with CSC, confirmed by optical coherence tomography (OCT) and fundus fluorescein angiography (FFA) were recruited from the Oxford Eye Hospital, John Radcliffe between 2012 and 2020. Sex, age, and visual acuity were recorded at the time of recruitment. Best corrected visual acuity (BCVA) of the better eye was utilised in this study. These same data were collected on 247 control participants without eye disease. As CSC has been associated with steroid use [22], this was recorded for participants. Of these, 5% (N = 5) of CSC patients and 2% of controls (N = 7) were currently utilising inhaled, topical, or oral steroids. These participants were not excluded from the study. This study obtained full approval in June 2011 through the Oxfordshire Research Ethics Committee (IRAS 43176, REC – South Central Oxford B 11/SC/0093) and was performed according to the tenets of the declaration of Helsinki (IRAS 43176, REC – South Central Oxford B 11/SC/0093).

### Inclusion and exclusion criteria

All control participants were over the age of 18, with a best corrected visual acuity (BCVA) of 6/7.5 or better in both eyes and no other significant past ocular history. Participants were excluded if they had prior treatment with benzodiazepines, a diagnosis of organic, physical or psychiatric conditions, a previous head injury, alcohol or drug abuse in the past or pregnancy.

### Assessments

Each participant completed a Pittsburgh Sleep Quality Index (PSQI) questionnaire [58] to assess global sleep quality defined through the seven subscores: (1) subjective sleep quality; (2) sleep latency; (3) sleep duration; (4) sleep efficiency; (5) sleep disturbance; (6) use of sleep medication and (7) daytime dysfunction. The Hospital Anxiety and Depression Scale (HADS) questionnaire was completed to assess anxiety and depression [59], Global Health Questionnaire (GHQ) to assess general health and use of medications, Epworth Sleepiness Scale (ESS) to assess daytime sleepiness [60] and the Morningness-Eveningness Questionnaire (MEQ) as well as morning and evening-type (MET) to explore chronotype and diurnal preference [61]. Poor sleep quality was defined as a PSQI > 5 [62], significant depression as a HADS-D 15–21, and significant anxiety as a HADS-A 15–21 (NICE Guidelines, 2023).

### Statistical analyses

Initial statistical analyses were performed using GraphPad Prism. Data were tested for normality using Shapiro-Wilk and Kolmogorov-Smirnov tests. Where data were not normal (as in comparisons between control and CSC cohorts) Mann-Whitney U tests or Kruskal-Wallis tests (when exploring sex-related effects) were used. Simple correlation analyses using Spearman’s R were used to explore the effects of age on sleep and HADS and MEQ scores. Unless otherwise stated, data are reported as mean ± standard deviation (SD). In addition, we performed linear model analyses in R Studio (Version 2022.02.2 + 485, R Core Team, 2023) to control for sex effects; these replicated our findings in GraphPad Prism and are thus not reported here.

## Results

### Demographics

247 controls (52 ± 13 years old) and 109 participants with CSC (54 ± 12 years old) were included in this study. Controls were age matched to CSC participants, with an age range of 30 – 83 years; no significant difference in age was seen between cohorts (U = 12531, *P* = 0.2986). Demographics are shown in detail in Table 1. No significant difference between cohorts for body mass index (BMI) (U = 12179, *P* = 0.791), general health (U = 12273, *P* = 0.312) emotional well-being (EWB) (U = 13055, *P* = 0.904) were identified. We also found no significant correlation in our cohorts with age and global PSQI, or HADS (*P* > 0.05). However, MEQ scores correlated with age (*P* < 0.001); suggesting with age individuals become more of a “morning-type”. In addition, a significance difference between sex (U = 8577, *P* < 0.0001), with a smaller proportion of females in the CSC group; which is to be expected given the greater prevalence of this condition in males.

**Table 1:** Demographics across control and CSC cohorts.

	Controls	CSC		
	All Patients	All Patients	Chronic	Acute
N (male/female)	247 (69/178)	109 (70/39)	88 (57/31)	21 (13/8)
Age years (mean, SD)	52 (13)	54 (12)	56 (11)	49 (12)
BCVA				
Mean, SD	N/A	0.060 (0.253)	0.0823 (0.266)	‒0.0280 (0.152)1
Missing	N/A	1	0	
BMI (mean, SD)	27.0 (6.92)	26.2 (5.94)	26.2 (6.10)	25.7 (5.28)
Missing	10	3	1	1
Global PSQI	5.59 (3.24)	5.26 (3.37)	5.32 (3.60)	4.90 (2.02)
Sleep quality	1.081 (0.739)	1.08 (0.73)	1.09 (0.748)	1.05 (0.686)
Sleep latency	0.773 (0.719)	0.648 (0.674)	0.671 (0.690)	0.600 (0.598)
Sleep duration	0.709 (0.853)	0.704 (0.889)	0.739 (0.941)	0.500 (0.607)
Sleep efficiency	0.761 (1.01)	0.824 (0.994)	0.852 (1.034)	0.600 (0.754)
Sleep disturbance	1.33 (0.551)	1.21 (0.513)	1.23 (0.541)	1.15 (0.366)
Sleep medication	0.174 (0.617)	0.157 (0.614)	0.171 (0.665)	0.050 (0.224)
Daytime dysfunction	0.757 (0.753)	0.657 (0.787)	0.602 (0.796)	0.950 (0.686)
ESS	6.37 (4.21)	5.78 (3.91)	5.89 (3.98)	5.53 (3.55)
HADS	8.58 (6.04)	8.14 (5.20)	8.30 (5.37)	7.53 (4.39)
HADS-A	5.52 (3.61)	5.37 (3.42)	5.39 (3.52)	5.21 (2.97)
HADS-D	3.05 (3.03)	2.76 (2.63)	2.90 (2.70)	2.32 (2.34)
MEQ (chronotype)	60.4 (9.41)	62.3 (8.96)	62.4 (9.01)	62.7 (9.21)
MET (diurnal preference)	1.59 (0.983)	1.39 (0.979)	1.38 (0.975)	1.37 (1.06)
Physical function	84.7 (21.6)	90.1 (16.2)	88.9 (17.4)	95.0 (7.07)
Emotional wellbeing	76.6 (16.5)	77.7 (14.2)	79.00 (14.3)	72.00 (12.2)
Social functioning	88.1 (19.8)	90.1 (19.9)	89.9 (19.4)	89.5 (22.5)
Pain	80.5 (22.0)	83.8 (22.1)	84.0 (22.4)	83.6 (21.1)
General health	70.9 (19.7)	72.9(18.4)	72.2 (18.1)	76.3 (19.9)

Here, other than for male/female, standard deviation (SD) is represented in brackets beside mean values. Best visual acuity denoted as “BCVA”.

Within the CSC group, 88 (81%) were cCSC and 21 (19%) aCSC. Within CSC cohorts, patients with cCSC exhibited a mean BCVA of 0.082 ± 0.27, age of 55.8 ± 11.2, and M:F sex ratio of 31:57, whereas patients with aCSC showed a BCVA of −0.028 ± 0.15, age of 48.6 ± 11.9 and M:F sex ratio of 8:13 (Table 1).

### Impacts of CSC

#### Sleep quality

**Fig. 1:**
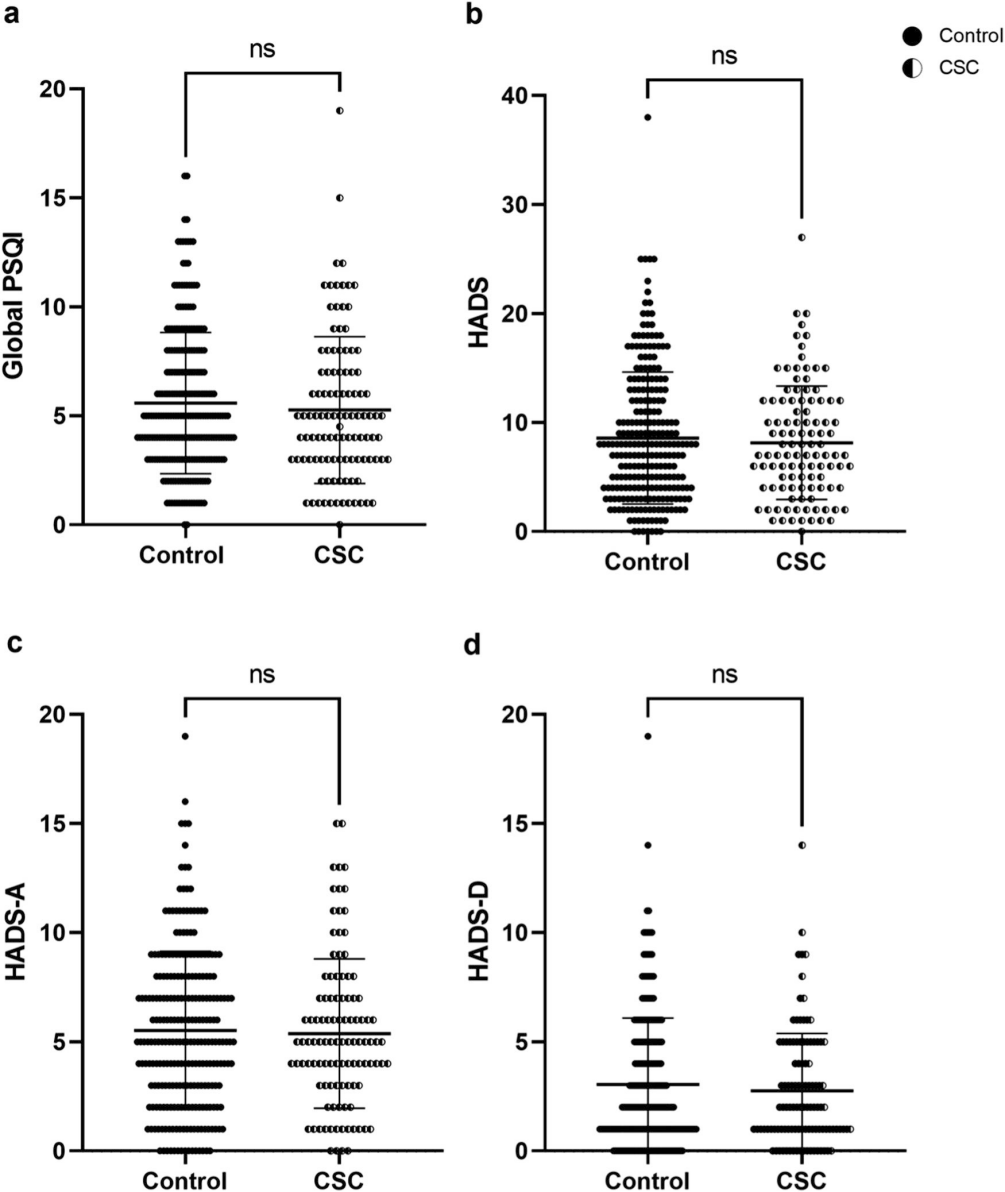
**Scatterplots showing the distribution of sleep and mood scores between control (filled circles,** ***N*** = **247) and CSC (half-moon circles,** ***N*** = **107) patients. a** Global Pittsburgh Sleep Quality Index (PSQI). **b** Hospitalised Anxiety and Depression Scale (HADS). **c** Depression (HADS-D). **d** Anxiety (HADS-A). Statistical analyses used were Mann-Whitney U testing. Data are shown as individual values with SD bars. “ns” denotes no significance.

No significant difference was found for PSQI (Mann-Whitney U = 12434, *P* = 0.307) between the control group (5.59 ± 3.24) and CSC patient group (5.26 ± 3.37) (Fig. 1a), nor between individual component scores for any parameter, nor chronotype or diurnal preference (*P* > 0.05; Table 2).

**Table 2:** Statistical analyses results from Mann-Whitney U testing, between controls, CSC and acute vs chronic (aCSC; cCSC), showing p-values for global PSQI and subscale components, as well as daytime dysfunction, sleepiness (ESS), Morning-eveningness questionnaire (MEQ) and MET revealing chronotype and diurnal preference.

P-values for PSQI, subscores and additional sleep measures	Controls vs CSC	aCSC vs cCSC
Global PSQI	0.307	0.845
Quality	0.938	0.912
Latency	0.141	0.793
Duration	0.828	0.506
Efficiency	0.529	0.475
Disturbance	0.062	0.497
Medication	0.657	0.722
Daytime dysfunction	0.151	0.0176*
ESS	0.305	0.729
MEQ: chronotype	0.110	0.953
MET: diurnal preference	0.097	0.993

*P < 0.05.

#### Sleep compared in aCSC and cCSC groups

As the natural history of chronic and acute CSC differ [33], we compared data within the two CSC groups. No difference for PSQI was identified nor for subscores except daytime dysfunction, which was significantly decreased in the cCSC group (Table 2). No correlation between aCSC and cCSC groups was identified when evaluating their PSQI as well as ESS, chronotype and diurnal preference (*P* > 0.05).

#### Anxiety and depression measures (indicators for mood)

To explore the effect of CSC on depression and anxiety, groups were compared for overall HADS (Fig. 1b). This revealed no significant differences between controls (8.58 ± 6.04) and CSC patients (8.14 ± 5.20), (U = 12999, *P* = 0.807). Anxiety and depression sub-scores (HADS-A, HADS-D) were also compared between groups (Fig. 1c, d). No significant differences were found for HADS-A both between control (5.52 ± 3.62) and CSC (5.37 ± 3.42), (U = 12849, *P* = 0.678), and nor for HADS-D (control: 3.05 ± 3.03, CSC: 2.76 ± 2.63; U = 12781, *P* = 0.619).

#### Anxiety and depression compared in the cCSC and aCSC groups

No significant difference in HADs was identified between aCSC and cCSC patients (U = 785.5, *P* = 0.684) nor HADS-A (U = 831, *P* = 0.969) or HADS-D (U = 736.5, *P* = 0.415) (data not shown). No significant correlations were found between chronicity and HADS (r = 0.0519, *P* = 0.597).

### Impact of sex and age on sleep and mood across groups

#### Sex

Given significant differences between sex ratios in our cohorts, we subcategorised into four groups: (i) control males (“control M”); (ii) control females (“control F”); (iii) CSC males (“CSC M”) and (iv) CSC females (“CSC F”).

**Fig. 2:**
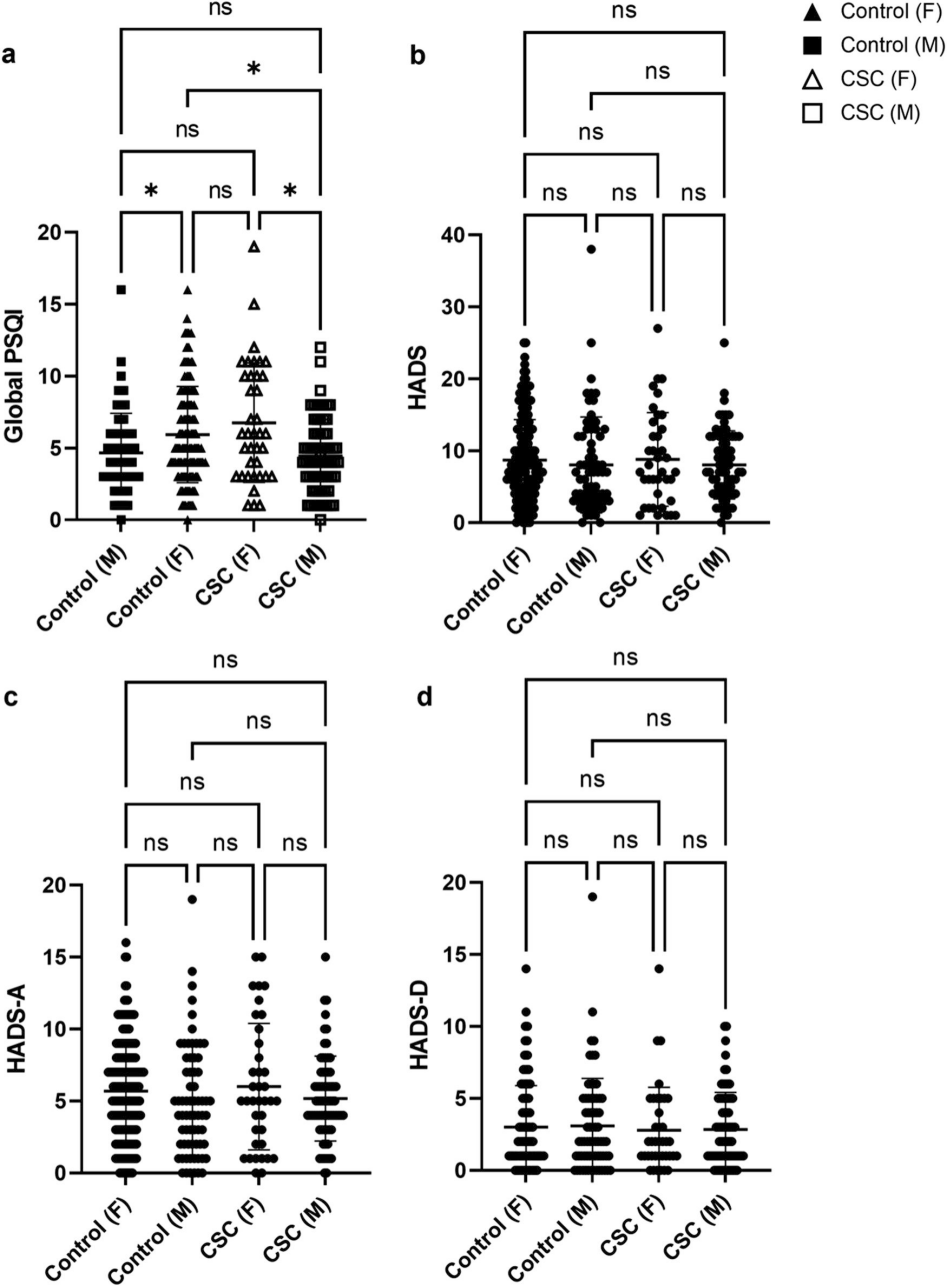
**Scatterplots showing the distribution of mood scores between (i) control females (**“**control F**”, **filled-in triangles,** ***N*** = **178); (ii) control males (**“**control M**”, **filled-in squares,** ***N*** = **68); (iii) CSC females**(“**CSC F**”**, empty triangles,** ***N*** = **38) and (iv) CSC males (**“**CSC M**”, **empty squares,** ***N*** = **70).** **a** Global Pittsburgh Sleep Quality Index (PSQI). **b** Hospitalised Anxiety and Depression Scale (HADS). **c** Depression (HADS-D). **d** Anxiety (HADS-A). Statistical analyses used here were Kruskal-Wallis testing, with Dunn’s post-hoc comparisons. Data are shown as individual values with SD bars. “ns” denotes no significance, * = *P* < 0.05.

A significant difference was seen between males and females for global PSQI (see Fig. 2a; K = 16.3, *P* = 0.001). Post hoc testing (Dunn’s multiple comparisons) showed a significant difference between CSC males compared to CSC females (*P* < 0.05; see Table 3). These data suggest that females exhibit worsened sleep both in control and CSC groups, when compared to males. No differences for HADS, HADS-A and HADS-D scores were observed (*P* > 0.05) (see Fig. 2b–d).

**Table 3:** Dunn’s multiple comparison post hoc testing between males and females for global PSQI.

Dunn’s multiple comparisons test	*P*-value
Control (F) vs Control (M)	0.048
Control (F) vs CSC (F)	>0.99
Control (F) vs CSC (M)	0.015
Control (M) vs CSC (F)	0.05
Control (M) vs CSC (M)	>0.99
CSC (F) vs CSC (M)	0.024

## Discussion

### Sleep

As in previous studies we used the PSQI to determine the effect of CSC on sleep. However, we did not identify any significant difference between control and CSC age-matched groups in our study. This contrasts with previous research by Ji et al. 2019, which examined age and sex-matched controls and showed a significant impact of CSC on sleep, with ~24% of their control patients exhibiting a PSQI of >5 [57]. In our control group, ~42% of our control population exhibited this (versus 38% with CSC). However, they did not report the mean of their control population. We compared our mean PSQI score (5.6), to previous literature with: (1) 4.4 (male) and 5.1 (female) in an American population [58]; (2) in a German population 36% of patients scored >5 on PSQI and; (3) a systematic review on PSQI scores reported a number of control groups averaging >5 [63]. Therefore, it is worth considering how sleep may differ in various cultures; as Ji et al. 2019, performed their study on a Chinese population we may be seeing different societal effects in controls.

#### Sex

In accordance with previous research, females exhibited worse sleep than males [64], than controls, and between CSC groups. Worsened sleep in females may become further exacerbated with age, due altered levels of oestrogen and progesterone [65]. A previous study also reported men to exhibit better general and mental health, with reduced role limitations when compared to women with CSC (mean age of 56 ± 10.05 years) [66]; we did not note this in our study (data not shown). When comparing age between groups, in our study no significant difference for age between CSC males vs females was identified (*P* > 0.999, Dunn’s multiple comparisons test,). However, females in our CSC group were significantly older than female controls (*P* = 0.0017, Dunn’s multiple comparisons test); as there is a complex interplay between the menopause, hormones and sleep [67], this could account for the lack of significance between CSC and control females.

#### Chronic versus acute

The influence of CSC chronicity, (which was not assessed previously [57]) was investigated in this study, and showed aCSC participants, when compared to cCSC, exhibit increased daytime dysfunction. cCSC is characterised by damaged retinal pigment epithelium and retina [31, 68, 69], versus temporary ( ~ 4 months; although may be recurrent) reduced visual acuity in aCSC is generally not expected to lead to retinal damage [70]. Given the numerous terms for CSC (acute, chronic, persistent, recurrent, subclinical or inactive diffuse epitheliopathy among others), with inherent inconsistencies in classification, strict categorisation of these two disorders is not always possible. It is possible that a more granular classification enabling further subgroup analysis would be beneficial to identify differences, and potential driving factors [71]. Given the proposed lack of RPE damage in aCSC, the impact on daytime dysfunction may be a result of short-term maladaptation due to reduced visual acuity.

#### Other influences

Of note, endogenous corticosteroid disruption, through the physiological response following sleep deprivation, has been associated with altered circadian rhythm modulation [72]. Elevated endogenous cortisol levels are reported in patients with CSC [73, 74], and concurrent CSC is reported in patients with Cushing syndrome [75, 76]. As previously mentioned, the use of exogenous steroids, irrespective of the route and timing, is known to precipitate or exacerbate CSC [43, 77, 78], as well as have a detrimental effect on sleep [65]. Indeed, half of CSC patients reported in one study have reported corticosteroid usage in the past [37]. This may suggest that SCRD occurring in individuals with CSC, may be potentially due to these precipitating, or secondary, factors. Our CSC cohort only contained patients with minimal steroid use (*N* = 5 out of 109 CSC patients; *N* = 7 out of 247); versus Ji et al. who completely excluded patients using steroids [57]. Furthermore, previous reports indicate that between 22 and 66% of CSC patients may have OSA [52–56], which is known to affect sleep and thus circadian rhythms. As our selection criteria also excluded systemic disease, it is possible the lack of sleep disturbance found in our study may be influenced by loss of these risk factors.

### HADS

For HADS overall, and HADS-A/D, no differences were revealed in our study between patients with CSC and controls. This contrasts with literature which highlights an association between CSC and a “type A” personality [40–42] and elevated levels of depressive, paranoic, obsessive, compulsive conditions and anxiety [79]. Anxiety has been speculated by Conrad et al. to be due to a higher incidence of physical complaints in CSC patients as well as inadequate coping strategies [41, 79]. Bazzazi et al. also demonstrated that anxiety was independent of first or recurrent disease [80]. CSC has also been reported to have an overall poorer quality of life when assessed with the symptom checklist 90-R and short form-36 [79, 81], however, we did not find this to be the case between our patient groups (*P* > 0.05). Studies in army veterans have shown PTSD as a factor contributing to CSC development [48], and in turn is linked to sleep disorders [82]. Our exclusion of patients with systemic disease and/or previous psychiatric conditions (e.g., depression, anxiety, and PTSD), may explain the difference between our results and previous literature.

#### Sex

Despite reports of men exhibiting improved general and mental health [66], we found no significant difference between males and females in HADS scores; this is supported by Bazzazi et al. who showed that women did not have higher anxiety scores than males. In patients suffering from idiopathic central serous CSC, anxiety scores are higher than healthy controls but do not change whether male or female, or with recurrent CSC [80].

#### Scope of the study and its limitations

The study we report here adds further information to this relatively unexplored field [57]. Chronic and acute forms of CSC were compared and their impacts on sleep and mood in an UK population– showing increased daytime dysfunction in aCSC patients. This has not previously been investigated, and highlights that aCSC has a differing impact on sleep, despite being a subtype not typically associated with retinal damage. We also explored daytime sleepiness, chronotype and diurnal preference as well as the effects of the sex of the individual.

This study was limited in that our participant controls exhibited PSQI > 5, suggesting poor sleep versus a lower proportion of controls with > 5 PSQI in [57]. However, this is reflective of other populations in Germany and America as well as noted in a systematic review of the literature [57, 58, 63].

For this study, as previously mentioned, we recruited more control participants compared to the number of CSC females, and more CSC males than CSC females. However, despite Ji et al. 2018 [57] reporting sex matching, their cohort exhibited a high proportion of males to females (110 males to 24 females in CSC and control groups). In our study females were shown overall to exhibit worse PSQI scores – this was not investigated by Ji et al. 2018. In view of this observation, and that the power of our study is affected by having fewer females in the affected group, we would recommend exploring the effects of the individual’s sex in CSC presentation using larger sex matched groups. This would enable a more in-depth analysis of the differences between acute and chronic presentations of this disease in males and females.

## Conclusion and future direction

The chronic form of CSC has deleterious effects on the retina, that may in turn damage the pRPGs which play a key role in calibrating our circadian rhythms. However, in this study we found no significant difference in sleep, anxiety or depression, between individuals with CSC and controls, which differs from the previous literature [57]. Within CSC grouping, aCSC participants exhibited increased daytime dysfunction, which could be driven through short term maladaptation during an acute episode. For future studies, we suggest: (i) polysomnography (PSG) as an objective measure; (ii) larger cohorts of well characterised aCSC and cCSC for comparison and (iii) age matched CSC female studies.

## Summary

### What was known before:

- Central Serous Chorioretinopathy (CSC) has been reported to impede sleep quality; but not yet studied in the UK.
- Chronic vs acute, as well as mood and gender had not been previously explored.

### What this study adds:

- First study in the UK exploring sleep and mood in CSC - overall suggests no significant difference.
- Within CSC groups worsened daytime function was observed in aCSC versus cCSC. A larger study would help distinguish the effect of aCSC versus cCSC on sleep.
- Worsened sleep for females was present when compared to males, both in CSC and control groups. Overall suggesting chronic and acute CSC patients, as well as females with CSC may be differentially impacted by the disease and further research would be useful in identifying what factors drive this.

## Data Availability

The data that support the findings of this study are not openly available due confidentiality, however, are available on request and stored at Oxford University Hospitals ERGO database.
